# Psychometric properties of the Chinese version of the trust between People and Automation Scale (TPAS) in Chinese adults

**DOI:** 10.1186/s41155-022-00219-x

**Published:** 2022-05-30

**Authors:** Jie Cai, Qian Sun, Zeyue Mu, Xiaoning Sun

**Affiliations:** 1 Department of Psychology, Zhejiang Sci-Tech University, Hangzhou, China; 2 School of Psychology and Cognitive Science, East China Normal University, Shanghai, China; 3 Social, Economic and Organisational Psychology Unit, Institute of Psychology, Leiden University, Leiden, The Netherlands; 4 School of Mental Health, Bengbu Medical College, Bengbu, China; 5 Department of Developmental and Behavioral Pediatrics, Pediatric Translational Medicine Institution, Shanghai Children’s Medical Center, School of Medicine, Shanghai Jiao Tong University, Shanghai, China

**Keywords:** Trust in automation, Interpersonal trust, Psychometric properties

## Abstract

Trust in automation plays a leading role in human-automation interaction. As there lack of scales measuring trust in automation in China, the purpose of this study was to adapt the trust between People and Automation Scale (TPAS) into Chinese and to demonstrate its psychometric properties among Chinese adults. A total of 310 Chinese adults were randomly selected as sample 1, and 508 Chinese adults as sample 2. Results of the item analysis revealed that each item had a good quality, and the exploratory factor analysis (EFA) and confirmatory factor analysis (CFA) suggested that the two-factor model with 12 items was the best fitting model. In addition, the TPAS was positively correlated with Interpersonal Trust Scale (ITS), proving good evidence based on relations to other variables to support the TPAS. In sum, the study suggested that the Chinese version of the TPAS could be used as an effective tool to assess trust in automation in the Chinese context.

With the development of the global economy and technology, various automation systems, such as aviation, maritime operations, and motor vehicle operation, are developing rapidly (Lee & See, 2004). This rapid development in automation systems is accompanied with a growing number of automation system practitioners or users. As such, it demands human decisions on the optimal approach to interact with the automated systems (Merritt & Ilgen, 2008). Previous studies showed that trust plays a leading role in human-automation interaction research, and trust in automation has become a demanding topic of research in the fields of psychology, automation, and related fields (Lee & See, 2004).

Trust in automation refers to the attitude that an agent helps achieve an individual’s goals in a situation characterized by uncertainty and vulnerability (Lee & See, 2004). Existing studies have suggested trust in automation as a crucial human factor that impacts human reliance on automation (Hussein et al., 2020b) as well as a key factor in the human-automation mismatch (Case et al., 1999). Moreover, trust in automation is considered a major determinant of acceptance and use of these advanced technologies (Drnec et al., 2016; Dzindolet et al., 2003). Therefore, researchers have gradually begun to attach importance to research and practice of trust in automation.

With the development of China’s economy and technology, automation systems are widely used (Cheng, 2009; Ling et al., 2015), warranting the necessity of investigating trust in automation and practical work in China. However, to the best of our knowledge, limited research has been done on the topic of trust in automation in China, likely due to the lack of effective measurement tools to assess trust in automation (Wang et al., 2017). Western researchers, on the other hand, have developed several methods for measuring trust in automation in order to explore how an individual expresses their trust when interacting with automation systems. These methods include self-report, behavioral measurement, and physiological measurement. Given the easiness in usage, self-report has become the most commonly utilized measurement tool. For example, Jian et al. (2000) developed the Trust between People and Automation Scale (TPAS), which aims to measure the degree of trust in automation. Yagoda and Gillan (2012) developed the Human-Robot Interaction Trust Scale to measure trust when humans interact with robots. Additionally, Gold et al. (2015) developed the Trust in Automation Scale to assess the trust when people interact with automation systems in Germany. Therefore, the development of related scales has become an important foundation to develop applicable measurement tools in China.

The current study aimed to develop a Chinese version of the TPAS. The TPAS is one of the most widely used scales of measurements in human-automation interaction scenarios and has been proven with excellent psychometric properties in the Western culture (Gulati et al., 2019; Hussein et al., 2020a, b; Wei et al., 2020), making the TPAS well acknowledged by the majority of researchers ( Manchon et al., 2021; Feng et al., 2019). The TPAS adopted methods of word elicitation, questionnaire, and paired comparison to generate items, allowing researchers to directly assess trust between humans and automation. In particular, the most notable advantage of the TPAS is its measurement of the general propensity to trust automated systems, providing a baseline measurement for predicting individual trust in a specific automated system (Jian et al., 2000).

In order to provide further evidence for the psychometric properties of the Chinese version, the current study aims to use interpersonal trust as the criterion for measurement. Interpersonal trust is the intention to accept vulnerability based on a reciprocal expectation from the trustee (Rousseau et al., 1998). The reasons for taking interpersonal trust as a criterion are threefold. First, existing evidence showed that interpersonal trust and trust in automation represent situation-specific attitudes that are relevant only when something is exchanged in a cooperative relationship characterized by uncertainty (Hoff & Bashir, 2015). Such findings indicated a conceptual relationship between interpersonal trust and trust in automation, making interpersonal trust a valuable criterion variable to support the TPAS. Second, people’s trust in technological systems represents their trust in the designers of such systems to some degree (Parasuraman & Riley, 1997). Finally, interpersonal trust and trust in automation share some of the same neural mechanisms (Dimoka, 2010; Riedl et al., 2010), which lays the physiological foundation for interpersonal trust as a criterion.

In summary, the aim of the present study was to adapt the TPAS into Chinese. Psychometric properties of the Chinese version were then investigated, additionally with the interpersonal trust being used as evidence based on relations to other variables to provide a scientific basis for the Chinese version of the TPAS.

## Method

### Participants and procedure

Data collection occurred from July 2021 to August 2021. We used a Chinese platform (www.wjx.cn) for questionnaire data collection. The recruitment procedure and research protocol were approved by the Institutional Review Board of the **** (masked for anonymous review). All participants started the survey after reviewing and agreeing to the informed consent. All individual responses were analyzed in a de-identified manner. In addition, according to the power analysis for the two-tailed correlative relationship, at an alpha level of 1%, a power of 90%, an expected correlative effect size at 0.3, and at least 158 participants should be included. In conclusion, the sample size in this study had sufficient statistical power.

#### Sample 1

We randomly recruited 312 native Chinese speakers. Extreme data, operationalized as data beyond three standard deviations from the mean, were excluded. Finally, sample 1 consisted of 310 valid data (*M*_age_ = 22.38 years, *SD*_age_ = 4.46, *Skewness*_age_ = 3.15, *Kurtosis*_age_ = 12.74, *Range*_age_ = 16~50 years), which were used for item analysis and EFA. Table 1 presents the demographic characteristics.

**Table 1:** Demographic characteristic of sample 1 and sample 2

Variables		Sample 1 (*N* =310)		Sample 2 (*N* =508)	
		*N*	Percent (%)	*N*	Percent (%)
Gender	Male	173	55.81	284	55.91
	Female	137	44.19	224	44.09
Education	Less than secondary school	3	0.97	17	3.35
	Secondary school	16	5.16	39	7.68
	College	250	80.64	362	71.25
	Postgraduate or above	41	13.23	90	17.72

#### Sample 2

We randomly selected 514 native Chinese speakers (no overlap with sample 1; demographic characteristics see Table 1). The final sample 2 consisted of 508 participants (*M*_age_ = 25.02 years, *SD*_age_ =7.05, *Skewness*_age_ = 2, *Kurtosis*_age_ = 5.86, *Range*_age_ = 17~58 years) after excluding extreme data. CFA and correlation analysis were performed with Sample 2.

### Instruments

#### Trust between People and Automation Scale (TPAS)

The original version of the scale included 12 items. Participants rated these items based on their feeling of trust or impression of the system on a 7-point Likert scale (1 = “not at all” to 7 = “extremely”) while operating a machine (Jian et al., 2000). An average score of 12 items was calculated, with higher scores representing a higher level of trust between people and automation.

The translation/back translation work of this research was carried out from June to July, 2021, and the following steps were performed to translate the TPAS into Chinese. First, we translated the TPAS items into simplified Chinese. We followed the translation/back-translation procedure recommended by Regmi et al. (2010). We invited two psychology researchers to independently translate each TPAS item into simplified Chinese. Item translation as well as any necessary modifications were then discussed to determine a single translated version. The translated version of the TPAS was then back-translated into English by a professional psychologist with bilingual proficiency in both English and Chinese. The original and the back-translated versions were compared and discussed by all authors, additionally recruiting 10 participants (no overlap with sample 1 or sample 2 participants) to evaluate unclear items. The Chinese version of the TPAS was then finalized.

#### Interpersonal Trust Scale (ITS)

The interpersonal trust was assessed using the Interpersonal Trust Scale (ITS) which was developed by Rotter (1967). The ITS consists of 25 items (e.g., “We can be the Court of Justice to place,” “Seems to have hope for the future”). Participants rated each item on a 5-point Likert scale (1 = “Strongly Disagree” to 5 = “Strongly Agree”). The higher the mean score, the higher levels of interpersonal trust are. In this survey, the Cronbach’s *α* coefficient was .95, and the odd-even split-half reliability coefficient was .91.

### Data analysis

Data analyses were performed using IBM SPSS 26.0 and Amos 21.0. Item analysis, exploratory factor analysis (EFA), confirmatory factor analysis (CFA), correlation analysis, and reliability analysis were used. First, we performed item analysis using the independent samples *t* test and item-total correlation to assess the quality of items. After that, EFA was performed to explore the factor structure and CFA was performed to verify the factor structure. The goodness-of-fit of the CFA models was evaluated by chi-square/degree of freedom ratio (*χ*^2^/*df*)*,* comparative fit index (CFI), Tucker-Lewis index (TLI), root mean square error of approximation (RMSEA), and root mean squared residual (SRMR). Among these indicators, the *χ*^2^/*df* values of up to 3 are treated as acceptable, CFI and TLI value above .90 represents reasonable fit, and RMSEA values equal and SRMR values equal to or less than 0.08 are considered as acceptable (Hu & Bentler, 1999; Medsker, 1994). Third, we used Pearson product-moment correlation analysis to test the evidence based on relations to other variables. Finally, we assessed the reliability of the Chinese version of the TPAS by Cronbach’s alphas coefficient and the odd-even split-half reliabilities.

## Result

### Item analysis

First, we selected the participants with the highest 27% of the total scores as the high score group and the participants with the lowest 27% as the low score group (Kelley, 1939) and then performed a *t* test to examine the differences between the high and low score groups for each item. As presented in Table 2, the results showed that each item significantly differed between the high and low score groups. Furthermore, we calculated item-total correlations, results of which indicated that all items exceed the acceptable criterion of .30 (Nunnally & Bernstein, 1994). In other words, the results of the item analysis demonstrated the suitable quality of each item.

**Table 2:** Results of item analysis (sample1 *N* = 310)

Item	*M*	*SD*	*t*	I-T
1. The system is deceptive	4.19	1.99	–7.56^***^	.38^**^
2. The system behaves in an underhanded manner	3.58	1.77	–5.12^***^	.36^**^
3. I am suspicious of the system’s intent, action, or outputs	3.85	1.81	–7.96^***^	.43^**^
4. I am wary of the system	3.54	1.72	–5.19^***^	.36^**^
5. The system’s actions will have a harmful or injurious outcome	3.87	1.79	–5.85^***^	.35^**^
6. I am confident in the system	5.13	1.53	–9.11^***^	.54^**^
7. The system provides security	4.96	1.56	–8.67^***^	.51^**^
8. The system has integrity	4.89	1.60	–8.43^***^	.48^**^
9. The system is dependable	4.99	1.48	–9.61^***^	.54^**^
10. The system is reliable	5.00	1.48	–9.56^***^	.53^**^
11. I can trust the system	4.87	1.47	–10.55^***^	.53^**^
12. I am familiar with the system	4.61	1.70	–5.27^***^	.31^**^

*Note*: ^**^p < .01, ^***^p < .001; I-T denotes item-total correlation

### Exploratory factor analysis

We conduct an EFA using the principal component method with oblique rotation in this section. The Kaiser-Meyer-Olkin (KMO) value was .92, while the Bartlett’s test of sphericity showed *χ*^2^/df = 31.54, *p* < 0.001, thus indicating that this sample was suitable for conducting EFA. Subsequently, we used parallel analysis to examine factor structure, which is one of the most accurate methods for determining a number of factors (Hayton et al., 2004). The results of the parallel analysis showed that this sample supported a two-factor solution. Finally, two factors were extracted by the principal component method with oblique rotation, the eigenvalues of the two factors were 5.67 and 2.27, respectively. And seven items were loaded on factor 1 and five items loaded on factor 2. The total interpretation rate of variance was 66.14%. Table 3 presents the factor loadings for each item and the loadings of the items are all greater than .5 (in a range of .59 to .85).

**Table 3:** Results of exploratory factor analysis (sample1 *N* = 310)

Item	Factor 1	Factor 2
1. The system is deceptive	–.17	**.82**
2. The system behaves in an underhanded manner	–.17	**.75**
3. I am suspicious of the system's intent, action, or outputs	–.11	**.85**
4. I am wary of the system	–.19	**.80**
5. The system’s actions will have a harmful or injurious outcome	–.22	**.84**
6. I am confident in the system	**.79**	–.08
7. The system provides security	**.80**	–.19
8. The system has integrity	**.77**	–.22
9. The system is dependable	**.83**	–.15
10. The system is reliable	**.82**	–.16
11. I can trust the system	**.79**	–.15
12. I am familiar with the system	**.59**	–.36

### Confirmatory factor analysis

**Fig. 1:**
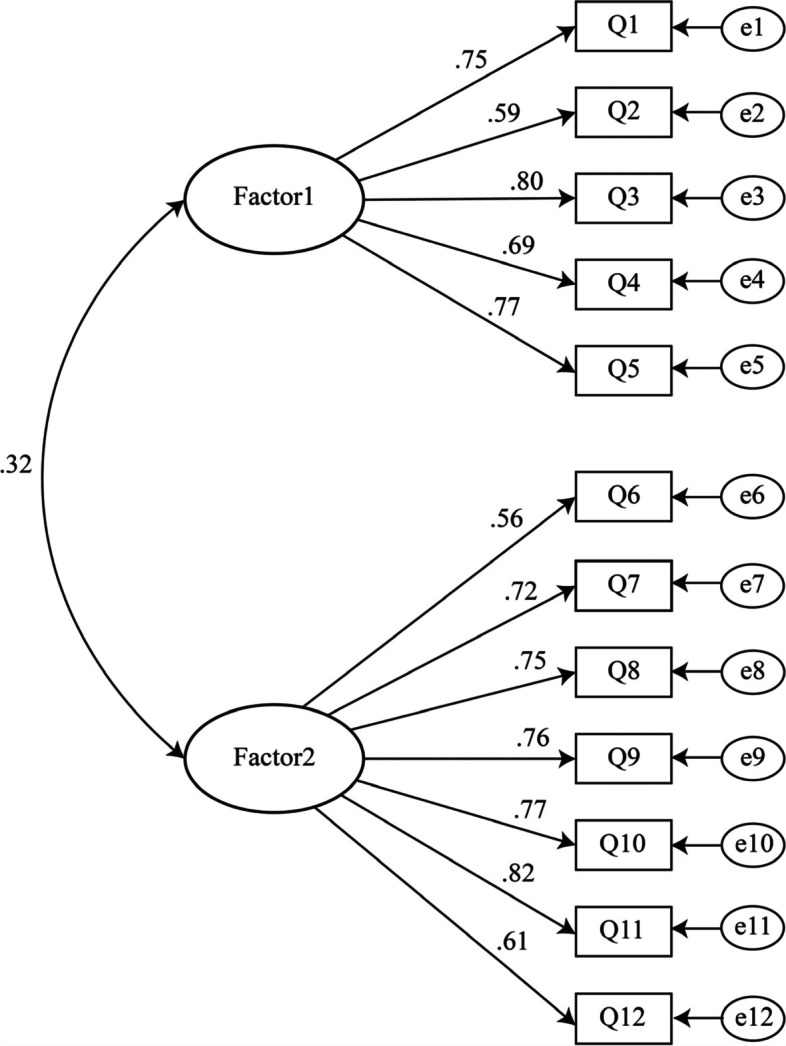
Standardized coefficient model of CFA

The result showed that all fitting indices met the statistical standards (see Table 4). The factor loadings were between .56 and .82, as shown in Fig. 1.

**Table 4:** Assessment of measurement models

Model	*χ*^2^	*df*	*χ*^2^/*df*	SRMR	CFI	TLI	RMSEA
Two-factor model	122.97	53	2.32	.05	.97	.97	.05

### Evidence based on relations to other variables

The results showed that the scale had a significantly positive correlation with the ITS average score (*r* = .22, *p* < 0.001), indicating good evidence based on relations to other variables for the Chinese version of the TPAS.

### Reliability analysis

The Cronbach’s alphas of the TPAS in samples 1 and 2 were .89 and .85, respectively. The odd-even split-half reliabilities of samples 1 and 2 were .85 and .87, respectively. These coefficients indicated that the scale had a good reliability.

## Discussion

The current study aimed to adapt the TPAS into Chinese and to demonstrate its psychometric properties among Chinese adults by investigating the item quality, factor structure, and evidence based on relation to other variables and reliability. In general, our findings were consistent with the original English version (Jian et al., 2000). Overall, the Chinese version of the TPAS is a valid and reliable measurement to assess trust in automation in China.

We conducted a series of analyses following the recommended steps of scale revision (Li et al., 2021; Ma & Liu, 2019), the results of EFA supported a two-factor structure of the TPAS, with each item having a high loading on its own factor and a low loading on the other factor. The total interpretation rate of variance was 66.14%, reaching the ideal criterion (Hair et al., 2012). This fully replicated the structure of the original English version of the TPAS (Jian et al., 2000). Moreover, the results of CFA confirmed a good fit of the two-factor model with 12 items, and all the factor loadings were significant, and correlations between two dimensions were in the expected direction, which was also consistent with the original scale (Jian et al., 2000). In addition, the evidence based on relations to other variables for the TPAS was tested via the relationship between the TPAS and the ITS. The result showed a significant positive correlation, indicating that the higher the individual’s trust in others, the higher the trust in automation. It is possible that trust in automation focuses on assessing the trust between human and automation, and it also represents their trust in the designers of such automation, indicating trust in automation can be viewed as a specific type of interpersonal trust (Hoff & Bashir, 2015). Together, the current results suggested that the Chinese version of the TPAS had high psychometric properties (Cortina, 1993), providing strong evidence of the Chinese TPAS being a reliable, effective, and culturally suitable measurement tool.

This study makes two key contributions to the literature. First, the Chinese version of the TPAS has good psychometric properties, providing an important research tool for research and practice of trust in automation in China. Given the previously reported significant cultural differences in trust in automation (Chien et al., 2016; Yerdon et al., 2017), our research will also be helpful for conducting cross-cultural comparative research to deepen the understanding of trust in automation. Second, interpersonal trust can be served as a valuable criterion to verify the effectiveness of trust in automation in the context of China. Previous studies have shown both interpersonal trust and trust in automation represent situation-specific attitudes that are relevant only when something is exchanged in a cooperative relationship characterized by uncertainty, which is the most fundamental reason for the correlation (Hoff & Bashir, 2015). However, previous studies were based on western culture, while there lacks related research in China. Our research revealed that interpersonal trust is also an effective criterion of trust in automation in Chinese culture.

Although this research has important strengths, several limitations should be addressed when interpreting the current findings. First, this study only assessed by one measurement tool (i.e., ITS) to provide evidence based on relations to other variables. Future studies should increase additional tools to deepen our understanding of the scale. Second, we only focused on Cronbach’s alpha and odd-even split-half reliability, other reliability indices (e.g., test-retest reliability of the scale) should be examined in the future. Third, most of our subjects were well-educated, possibly limiting the generalizability of our results. Additional research is needed to test the TPAS in more diverse subpopulations in China, such as those with lower education levels.

## Conclusions

The current study is among the first to adapt a trust in automation scale among Chinese adults and extend the applicability of the trust in automation scale to Chinese culture. The results indicate that the Chinese version of the TPAS is a valid and reliable tool for developing the research and practice of trust in automation in the Chinese context.

## Data Availability

The research materials that support the findings of this study are available on request from the corresponding author. The research materials are not publicly available due to privacy or ethical restrictions.

## References

[CR1] Case K, Sinclair MA, Abdul Rani MR (1999). An experimental investigation of human mismatches in machining. Proceedings of the Institution of Mechanical Engineers, Part B: Journal of Engineering Manufacture.

[CR2] Cheng D (2009). Advances in automation and control research in China. Science in China Series F: Information Sciences.

[CR3] Chien S, Lewis M, Sycara K, Liu J-S, Kumru A (2016). Influence of cultural factors in dynamic trust in automation. *2016 IEEE International Conference on Systems,Man, and Cybernetics(SMC)*.

[CR4] Cortina JM (1993). What Is Coefficient Alpha? An examination of theory and applications. Journal of Applied Psychology.

[CR5] Dimoka A (2010). What does the brain tell us about trust and distrust? evidence from a functional neuroimaging study. MIS Quarterly.

[CR6] Drnec K, Marathe AR, Lukos JR, Metcalfe JS (2016). From trust in automation to decision neuroscience: applying cognitive neuroscience methods to understand and improve interaction decisions involved in human automation interaction. Frontiers in Human Neuroscience.

[CR7] Dzindolet MT, Peterson SA, Pomranky RA, Pierce LG, Beck HP (2003). The role of trust in automation reliance. International Journal of Human Computer Studies.

[CR8] Feng J, Sanchez J, Sall R, Lyons JB, Nam CS (2019). Emotional expressions facilitate human–human trust when using automation in high-risk situations. Military Psychology.

[CR9] Gold C, Körber M, Hohenberger C, Lechner D, Bengler K (2015). Trust in automation – before and after the experience of take-over scenarios in a highly automated vehicle. Procedia Manufacturing.

[CR10] Gulati S, Sousa S, Lamas D (2019). Design, development and evaluation of a human-computer trust scale. Behaviour and Information Technology.

[CR11] Hair JF, Anderson RE, Tatham RL, Black WC (2012). *Multivariate data analysis* (7th Editio).

[CR12] Hayton JC, Allen DG, Scarpello V (2004). Factor retention decisions in exploratory factor analysis: a tutorial on parallel analysis. Organizational Research Methods.

[CR13] Hoff KA, Bashir M (2015). Trust in automation: integrating empirical evidence on factors that influence trust. Human Factors.

[CR14] Hu L, Bentler PM (1999). Cutoff criteria for fit indexes in covariance structure analysis: conventional criteria versus new alternatives. Structural Equation Modeling.

[CR15] Hussein A, Elsawah S, Abbass HA (2020). The reliability and transparency bases of trust in human-swarm interaction: principles and implications. Ergonomics.

[CR16] Hussein A, Elsawah S, Abbass HA (2020). Trust mediating reliability–reliance relationship in supervisory control of human–swarm interactions. Human Factors.

[CR17] Jian, J.-Y., Bisantz, A. M., & Drury, C. G. (2000). Foundations for an empirically determined scale of trust in automated systems. *International Journal of Cognitive Ergonomics, 4*, (1), 53–71 10.1207/s15327566ijce0401_04.

[CR18] Kelley TL (1939). The selection of upper and lower groups for the validation of test items. Journal of Educational Psychology.

[CR19] Lee JD, See KA (2004). Trust in automation: designing for appropriate reliance. Human Factors.

[CR20] Li, A., Wang, S., Paetzold, R. L., & Liu, X. (2021). Validity and reliability of the Chinese version of adult disorganized attachment scale in Chinese adults. *Current Psychology*, *1* Advance online publication.

[CR21] Ling W, Liu D, Yang D, Sun C (2015). The situation and trends of feeder automation in China. Renewable and Sustainable Energy Reviews.

[CR22] Ma J, Liu C (2019). Psychometric properties of the Chinese version of the social media burnout scale. Current Psychology.

[CR23] Manchon JB, Bueno M, Navarro J (2021). From manual to automated driving: how does trust evolve?. Theoretical Issues in Ergonomics Science.

[CR24] Medsker G (1994). A review of current practices for evaluating causal models in organizational behavior and human resources management research. Journal of Management.

[CR25] Merritt SM, Ilgen DR (2008). Not all trust is created equal: dispositional and history-based trust in human-automation interactions. Human Factors.

[CR26] Nunnally JC, Bernstein IH (1994). *Psychometric Theory*.

[CR27] Parasuraman R, Riley V (1997). Humans and automation: use, misuse, disuse, abuse. Human Factors.

[CR28] Regmi K, Naidoo J, Pilkington P (2010). Understanding the processes of translation and transliteration in qualitative research. International Journal of Qualitative Methods.

[CR29] Riedl R, Hubert M, Kenning P (2010). Are there neural gender differences in online trust? An fMRI Study on the Perceived Trustworthiness of eBay Offers. MIS Quarterly.

[CR30] Rotter JB (1967). A new scale for the measurement of interpersonal trust. Journal of Personality.

[CR31] Rousseau DM, Sitkin S, Burt RS, Camerer C (1998). Not so different after all: a cross-discipline view of trust. Academy of Management Review.

[CR32] Wang XY, Li Y, Chang M, You XQ (2017). The detriments and improvement of automation trust and dependence to aviation safety. Advances in Psychological Science.

[CR33] Wei J, Bolton ML, Humphrey L (2020). The level of measurement of trust in automation. Theoretical Issues in Ergonomics Science.

[CR34] Yagoda RE, Gillan DJ (2012). You Want Me to Trust a ROBOT? The development of a human-robot interaction trust scale. International Journal of Social Robotics.

[CR35] Yerdon VA, Marlowe TA, Volante WG, Li S, Hancock PA (2017). Investigating cross-cultural differences in trust levels of automotive automation. *Advances in Cross-Cultural Decision Making*.

